# Seven wood-inhabiting new species of the genus *Trichoderma* (Fungi, Ascomycota) in Viride clade

**DOI:** 10.1038/srep27074

**Published:** 2016-06-01

**Authors:** Wen-Tao Qin, Wen-Ying Zhuang

**Affiliations:** 1 State Key Laboratory of Mycology, Institute of Microbiology, Chinese Academy of Sciences, Beijing 100101, P.R. China; 2 University of Chinese Academy of Sciences, Beijing 100049, P.R. China

## Abstract

More than 200 recent collections of *Trichoderma* from China were examined and 16 species belonging to the Viride clade were identified based on integrated studies of phenotypic and molecular data. Among them, seven wood-inhabiting new species, *T. albofulvopsis*, *T. densum*, *T. laevisporum*, *T. sinokoningii*, *T. sparsum*, *T. sphaerosporum* and *T. subviride*, are found. They form trichoderma- to verticillium-like conidiophores, lageniform to subulate phialides and globose to ellipsoidal conidia, but vary greatly in colony features, growth rates, and sizes of phialides and conidia. To explore their taxonomic positions, the phylogenetic tree including all the known species of the Viride clade is constructed based on sequence analyses of the combined RNA polymerase II subunit b and translation elongation factor 1 alpha exon genes. Our results indicated that the seven new species were well-located in the Koningii, Rogersonii and Neorufum subclades as well as a few independent terminal branches. They are clearly distinguishable from any existing species. Morphological distinctions and sequence divergences between the new species and their close relatives were discussed.

Viride clade is the largest and most diverse group of the genus *Trichoderma* Pers. (Ascomycota, Sordariomycetes, Hypocreales). Species in this clade can be isolated from very diverse sources with a wide geographic distribution^1^. They are beneficial to industry, agriculture, medicine and other fields^2^,^3^. For example, *T. viride* well inhibits the decay of obeche (*Triplochiton sceleroxylon*) wood infected by *Gloeophyllum* sp. and *G. sepiarium* by means of mycoparasitism and competition for nutrients^4^. Nevertheless, some taxa, such as *T. asperellum* and *T. atroviride*, are regarded as threats to commercial mushroom production^5^ or even human health^6^.

The Viride clade was originally under the name “section Trichoderma” including the type species of the genus, *T. viride*^7^. Species in this clade mostly form brown to rufous stromata with inconspicuous ostiolar dots, hyaline ascospores, trichoderma-, verticillium- or pachybasium-like conidiophores with paired, verticillate phialides and green conidia. Their asexual states vary greatly in colony features, conidiophore branching patterns, growth rates, and conidial shapes and sizes^8^,^9^. High morphological homoplasy in sexual state makes identification difficult, which increases the importance of the characteristics of asexual state and sequence data. Based on the combined phenotypic and molecular data, Samuels *et al.* treated the *T. koningii* aggregate species as 12 taxonomic species and one variety^10^, and Jaklitsch *et al.* disentangled the *T. viridescens* complex by recognizing 11 independent species^11^. Concept of the clade has been renewed over time. Jaklitsch and Voglmayr renamed the clade as Viride clade and provided an updated comprehensive phylogenetic tree based on *RPB2* or *TEF1-α* sequences, in which six subclades were suggested^12^.

Early study of *Trichoderma* species in the Viride clade from China dates back to Teng when *T. viride* [as *Hypocrea rufa* (Pers.) Fr.] was recorded from Anhui, Hunan and Jiangsu provinces^13^. Later findings were attributed to Wen *et al.*^14^, Zhang & Xu^15^, Yu *et al.*^16^ and Zhu & Zhuang^17^. To give a better understanding of *Trichoderma* species diversity of the country, more recent collections were studied and seven new species belonging to the Viride clade were discovered. They are described, illustrated and named as *T. albofulvopsis*, *T. densum*, *T. laevisporum*, *T. sinokoningii*, *T. sparsum*, *T. sphaerosporum* and *T. subviride*. Their phylogenetic positions were also explored inferred from sequence analyses of the combined RNA polymerase II subunit b (*RPB2*) and translation elongation factor 1 alpha (*TEF1-α*) exon genes. Detailed comparisons were made between the new taxa and their related fungi.

## Results

### Phylogenetic analyses

**Figure 1:**
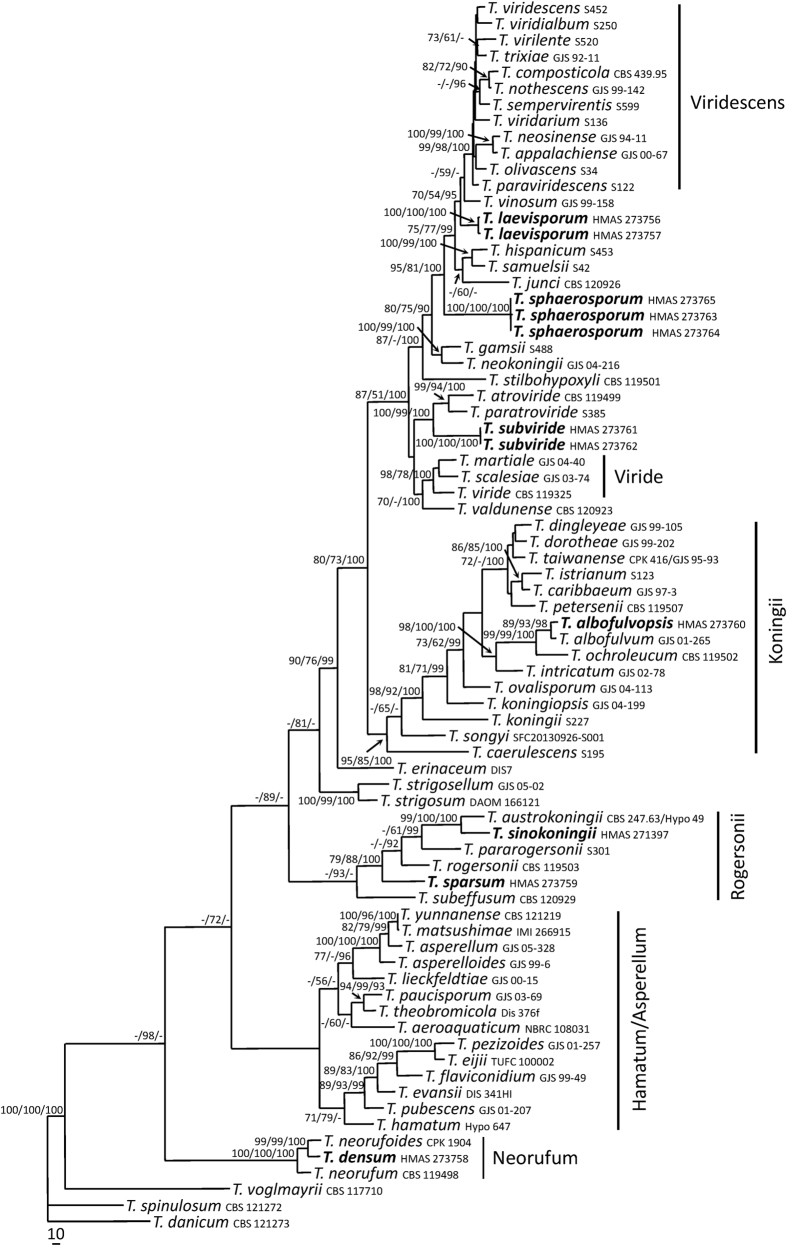
Maximum parsimony phylogram reconstructed from the combined sequences of *RPB2* and *TEF1-α*. MLBP above 70% (left), MPBP above 50% (middle), BIPP above 90% (right) are indicated at the nodes. New species proposed are indicated in boldface.

The partition homogeneity test (P = 0.01) of *RPB2* and *TEF1-α* sequences indicated that the individual partitions were generally congruent^18^. Phylogenetic positions of the new species were determined by analyses of the combined *RPB2* and *TEF1-α* data set containing 72 taxa and 2479 characters. Bayesian Inference (BI), Maximum likelihood (ML) and Maximum parsimony (MP) trees generated shared the same topology. In MP analyses, 1504 characters were constant, 207 variable characters were parsimony-uninformative, and 768 were parsimony-informative. One of the 76 most-parsimonious trees was shown here as Fig. 1 (Tree length = 3985, Consistency index = 0.3967, Homoplasy index = 0.6033, Rescaled consistency index = 0.2475, Retention index = 0.6238).

Seventy-four combined sequences of hyaline-ascospored species including our seven new species were used in the analyses with two green-ascospored species, *T. danicum* and *T. spinulosum*, as outgroup taxa. As shown in the phylogenetic tree (Fig. 1), all the hyaline-ascospored species investigated formed a strongly supported group (MLBP/MPBP/BIPP = 100%/100%/100%). Sixty-nine taxa of the Viride clade clustered together (MPBP = 98%) with six recognized subclades, Hamatum/Asperellum, Koningii (MLBP/MPBP/BIPP = 95%/85%/100%), Neorufum (MLBP/MPBP/BIPP = 100%/100%/100%), Rogersonii (MPBP/BIPP = 93%/89%), Viride (MLBP/MPBP/BIPP = 98%/78%/100%) and Viridescens (MLBP/MPBP/BIPP = 99%/98%/100%), as well as a few independent terminal branches. The tree topology is basically congruent with the previous reports^12^,^19^.

The seven new species proposed were scattered in three named subclades plus three unnamed terminal branches (Fig. 1). *Trichoderma albofulvopsis* and *T. albofulvum* (MLBP/MPBP/BIPP = 89%/93%/98%) and *T. densum* and *T. neorufoides* (MLBP/MPBP/BIPP = 99%/99%/100%) appeared as two sister pairs in the Koningii and Neorufum subclades respectively. Species in each sister pair shared common morphological features but were distinct at species level. *Trichoderma sinokoningii* and *T. austrokoningii* (MLBP/MPBP/BIPP = 100%/100%/100%) were also sisters, which share a common ancestor with *T. sparsum*, *T. rogersonii*, *T. pararogeronii* and *T. subeffusum* (MPBP/BIPP = 93%/89%) in the Rogersonii subclade. The two strains of *T. laevisporum* (MLBP/MPBP/BIPP = 100%/100%/100%) and the three ones of *T. sphaerosporum* (MLBP/MPBP/BIPP = 100%/100%/100%) were not closely related to any of the subclades. The two strains of *T. subviride* were associated with *T. paratroviride* and *T. atroviride*, and formed a highly supported terminal branch (MLBP/MPBP/BIPP = 100%/99%/100%).

## Taxonomy

### **Trichoderma albofulvopsis**

**Figure 2:**
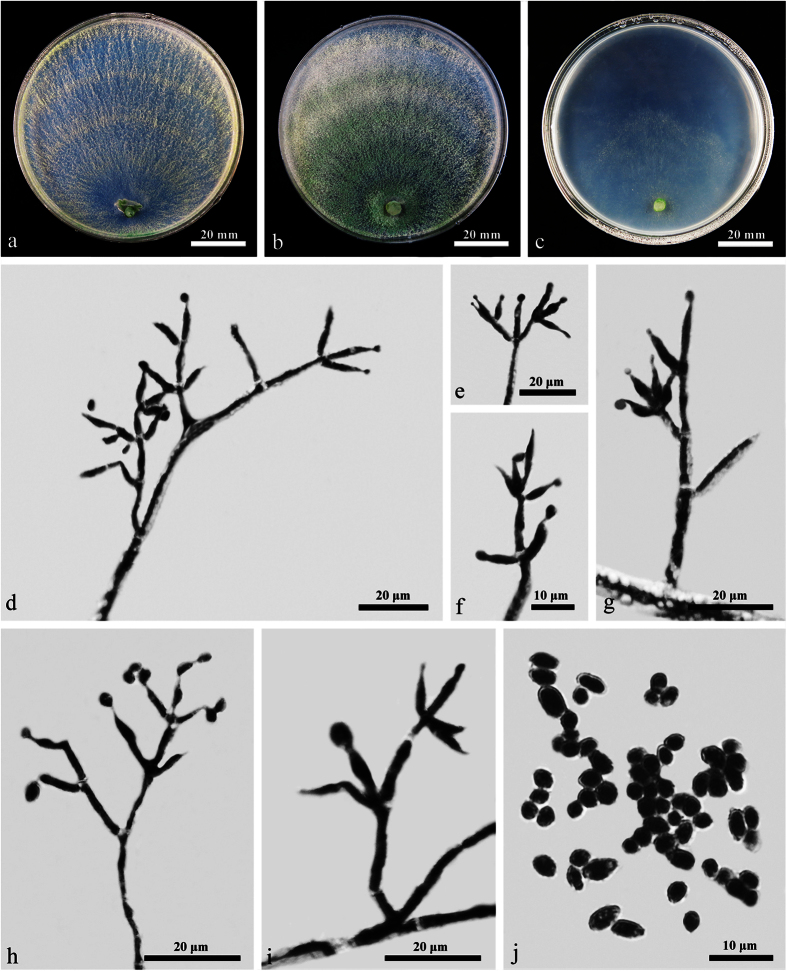
*Trichoderma albofulvopsis* (HMAS 273760). (**a–c**) Cultures after 6 d at 25 C (**a**: CMD, **b**: PDA, **c**: SNA); (**d–i**) Conidiophores and phialides (CMD, 5 d); (**j**) Conidia (CMD, 5 d).

W.T. Qin & W.Y. Zhuang, **sp. nov.** (Fig. 2) Fungal Names: FN570242

#### Typification

China. Hubei, Shennongjia, Banqiao, alt. 1455 m, on twigs, 20 Sep 2014, W.T. Qin, K. Chen, Z.Q. Zeng & H.D. Zheng 9930 (holotype HMAS 273760).

#### Etymology

The specific epithet refers to the similarity to *T. albofulvum*.

On CMD after 72 h 38–40 mm and mycelium covering the plate after 5 d at 25 C. Colony circular, with distinct denser/looser concentric zones and looser zones much wider. Surface downy, floccose or farinose, yellowish green to green. Conidiation noted after 3 d. No distinct odor, no diffusing pigment observed.

On PDA after 72 h 35–41 mm and mycelium covering the plate after 5 d at 25 C. Colony dense and zonate, aerial hyphae frequent, forming radial strands, green to pale green. Conidiation starting after 2 d on and around the plug, effuse, spreading slowly towards the distal margin. A distinct coconut-like odor detected, pigment inconspicuous or pale yellow.

On SNA after 72 h 15–22 mm and mycelium covering the plate after 12 d at 25 C. Colony hyaline, thin, with a well-defined or irregular margin. Conidiophores noted after 3 d, trichoderma-like, phialides solitary or commonly divergent in whorls of 2–4(–5), lageniform, subulate or slightly ampulliform, often subulate in the middle of the whorls, (5.5–)6–11(–12.4) × 2–3.2(–3.5) μm, l/w (2–)2.3–4.2(–4.4), 1.4–2.2 μm wide at the base (n = 40). Conidia light green or yellowish green, subglobose, globose or ellipsoidal, smooth, (2.8–)3.2–4.2(–4.8) × 2.5–3.2 μm, l/w (1.0–)1.4–1.5 (n = 50). No chlamydospores observed. Autolytic excretions rare, coilings frequent. No distinct odor, no diffusing pigment observed.

#### Notes

As the sister of *T. albofulvopsis* in the Koningii subclade (Fig. 1), *T. albofulvum* also forms trichoderma-like conidiophores, but the presence of reddish brown pigments in culture, fast-growth, and green smaller conidia [2.8–3.5 × 1.8–3.0 μm] apparently separate them. And the conidiophore branches of *T. albofulvum* are more complex, and phialides are solitary or commonly divergent in whorls of 2–5(–6)^20^. Compared with the sequences from the epitype of *T. albofulvum*^21^, similarities of *RPB2* and *TEF1-α* were 98.7% and 98.9%, and with 13 bp and 8 bp differences among 1019 bp and 729 bp, respectively.

*Trichoderma albofulvopsis* is similar to *T. ochroleucum* in conidia and conidiophores, but the latter is distinguished by different growth rates in cultures [CMD 29–31 mm, PDA 27–30 mm, SNA 25–27 mm], earlier sporulation [after 24 h]^19^, and the sequence data.

### **Trichoderma densum**

**Figure 3:**
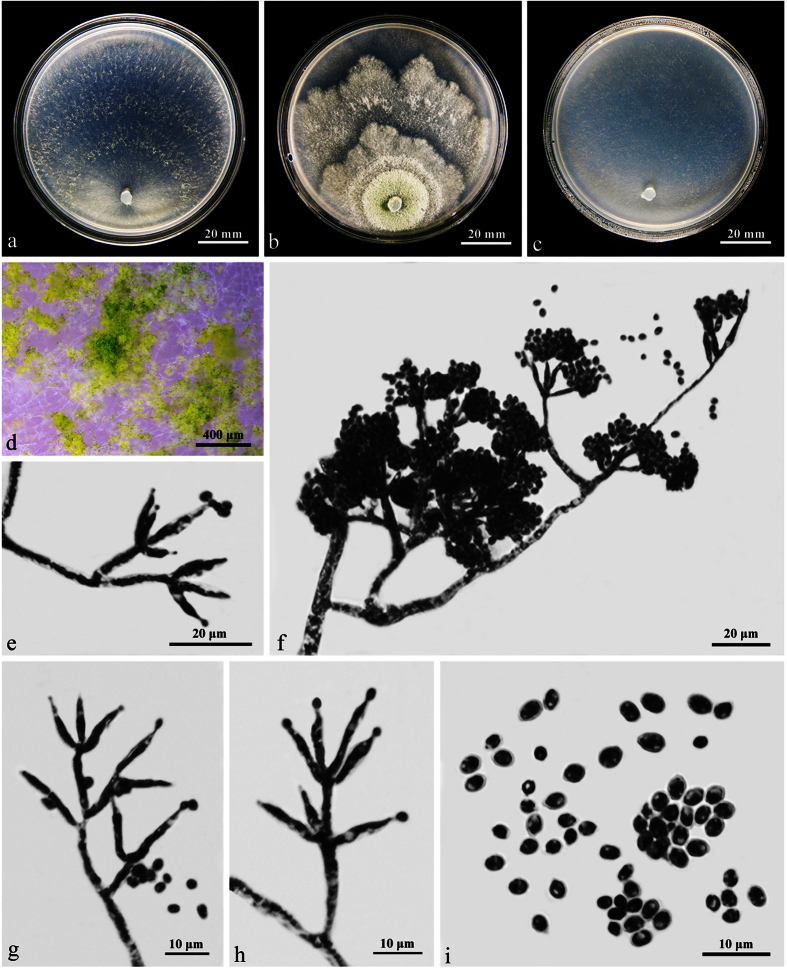
*Trichoderma densum* (HMAS 273758). (**a–c**) Cultures at 25 C (**a**: CMD, 5 d; **b**: PDA, 11 d; **c**: SNA, 11 d); (**d**) Conidiation pustules (CMD, 7 d); (**e–h**) Conidiophores and phialides (CMD, 5 d); (**i**) Conidia (CMD, 5 d).

W.T. Qin & W.Y. Zhuang, **sp. nov.** (Fig. 3) Fungal Names: FN570243

#### Typification

China. Beijing, Yanqing, Yudushan, alt. 906 m, on twigs, 27 Jun 2015, X.C. Wang, H.D. Zheng, Z.Q. Zeng, K. Chen & W.T. Qin 10165 (holotype HMAS 273758).

#### Etymology

The specific epithet refers to the densely disposed conidiophores and phialides.

On CMD after 72 h 40–42 mm and mycelium covering the plate after 5 d at 25 C. Colony hyaline, circular, with well-defined margin. Aerial hyphae numerous, becoming fertile toward the distal margin. Finely downy concentric zones produced by effuse conidiation. Conidiation noted after 2 d, conidiophores trichoderma- to verticillium-like, phialides solitary or commonly divergent in whorls of 2–4(–5), lageniform to subulate, (6–)8–18(–20) × (2–)2.5–3 μm, l/w (2.4–)3–6.4(–6.7), 1.8–2.8 wide at the base (n = 50). Conidia green, mostly ellipsoidal or oblong, less commonly subglobose, smooth, 2.8–4.5(–5) × 2.5–3(–3.5) μm, l/w 1.0–1.7(–2) (n = 50). Chlamydospores lacking or rare. No distinct odor, no diffusing pigment observed.

On PDA after 72 h 27–30 mm and mycelium covering the plate after 8 d at 25 C. Colony first hyaline, with coarsely wavy margin, not zonate; aerial hyphae numerous, thick, radially arranged on the margin, gradually forming a thick mat separated into 2–3 broad zones with irregular outline and a whitish to pale yellowish, downy to finely floccose surface. Conidiation noted after 24 h around the plug. Chlamydospores lacking or rare. A distinct coconut-like odor detected, no diffusing pigment observed.

On SNA after 72 h 32–35 mm and mycelium covering the plate after 7 d at 25 C. Colony homogeneous, not zonate, margin radially fan-shaped. Aerial hyphae sparser than that on CMD. Conidiation starting after 24–48 h, no chlamydospores observed. A faint coconut-like odor detected, no diffusing pigment observed.

#### Notes

As showed in Fig. 1, *T. densum*, *T. neorufoides* and *T. neorufum* formed the subclade Neorufum. Among them, the phialides of *T. densum* and *T. neorufoides* are similar in size, while the latter produces denser tufts or pustules on CMD and yellow, golden yellow to brownish pigments on PDA, grows slower in cultures [CMD 21–24 mm, PDA 24–26 mm, SNA 21–22 mm], and forms larger conidia [(3.4–)4.0–5.6(–7.4) × (2.3–)2.7–3.2(–3.8) μm]^19^. As to sequence divergences between the two fungi, 10 bp and 18 bp differences among 1029 bp and 1199 bp for *RPB2* and *TEF1-α* respectively were detected.

Compared with *T. densum*, *T. neorufum* grows slower in cultures [CMD 28–29 mm, PDA 23–25 mm, SNA 25–26 mm] and forms pachybasium-like instead of trichoderma- or verticillium-like conidiophores in abundant pustules on CMD and SNA. *Trichoderma neorufum* has yellowish to pale brownish irregularly serrate concentric zones on PDA and yellow to brown-orange pigments on CMD and PDA^19^, which are absent in *T. densum*.

### **Trichoderma laevisporum**

**Figure 4:**
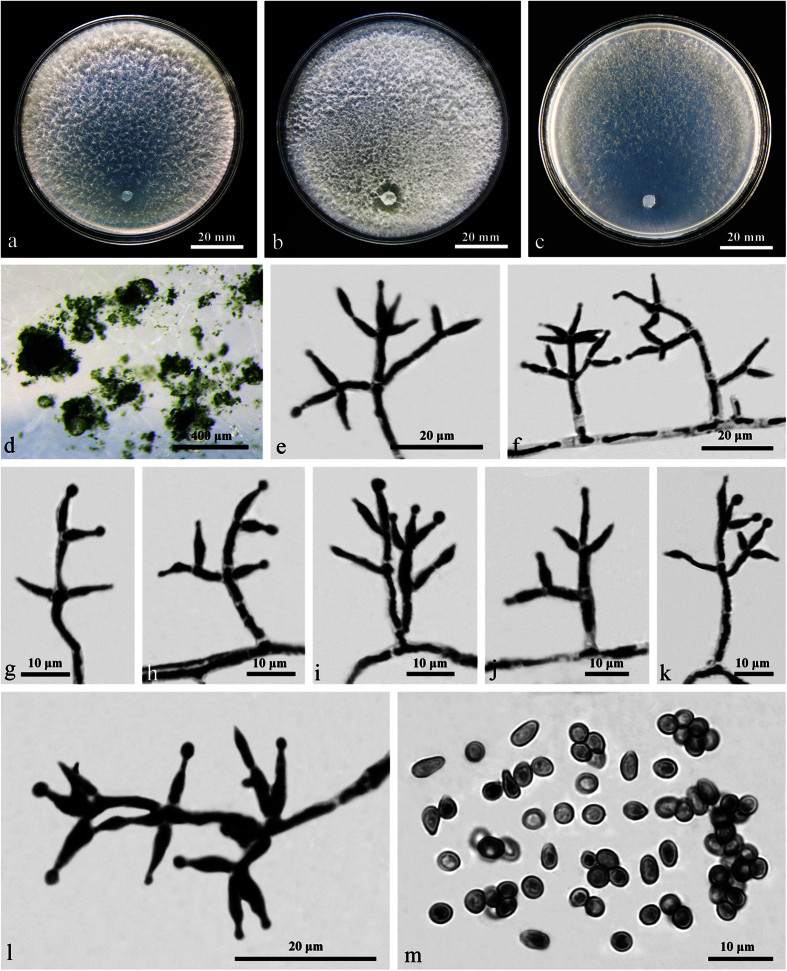
*Trichoderma laevisporum* (HMAS 273756). (**a–c**) Cultures at 25 C (**a**: CMD, 17 d; **b**: PDA, 17 d; **c**: SNA, 9 d); (**d**) Conidiation pustules (SNA, 17 d); (**e–l**) Conidiophores and phialides (SNA, 9 d); (**m**) Conidia (SNA, 9 d).

W.T. Qin & W.Y. Zhuang, **sp. nov.** (Fig. 4) Fungal Names: FN570248

#### Typification

China. Hubei, Shennongjia, Dalongtan, alt. 2000 m, on twigs, 13 Sep 2014, W.T. Qin, Z.Q. Zeng, H.D. Zheng & K. Chen 9481 (holotype HMAS 273756).

#### Etymology

The specific epithet refers to the smooth conidia.

On CMD after 72 h 48–50 mm and mycelium covering the plate after 5 d at 25 C. Colony dense, whitish, surface downy, farinose to floccose, with marginal surface hyphae thicker. Conidiation noted after 4 d. A distinct coconut-like odor detected, no diffusing pigment observed.

On PDA after 72 h 43–45 mm and mycelium covering the plate after 5 d at 25 C. Colony similar to but denser than that on CMD, macroscopically homogeneous, whitish, surface downy, farinose to floccose, covered by abundant aerial hyphae several mm thick. Conidiation noted after 3 d. A distinct coconut-like odor detected, no diffusing pigment observed.

On SNA after 72 h 33–35 mm and mycelium covering the plate after 6 d at 25 C. Colony dense, whitish to grayish white, radially fan-shaped. Aerial hyphae conspicuous, with marginal surface hyphae thicker than that around the plug. Conidiophores trichoderma-like, noted after 4 d, with 2–4(–5) whorls arising from the main axis. Phialides mostly symmetrically arranged, straight, narrowly lageniform or subulate, (6–)6.5–12(–14) × 2–3 μm, l/w 2.5–4.6(–6.4), 1.5–2.2 μm wide at the base (n = 80). Conidia green, mostly subglobose or ellipsoidal, sometimes ovoid or pyriform, smooth, (2.5–)3–5.5(–6) × (2–)2.5–3.5(–3.8) μm, l/w 1.0–1.8(–2.7) (n = 80). Chlamydospores noted after 8 d, terminal and intercalary, globose or ellipsoidal, 5–11 × 3–10 μm, l/w 1.0–1.4(–1.6) (n = 80). A distinct coconut-like odor detected, no diffusing pigment observed.

#### Other specimen examined

China. Hubei, Shennongjia, Tianziya, alt. 2000 m, on twigs, 16 Sep 2014, W.T. Qin, K. Chen, H.D. Zheng & Z.Q. Zeng 9715, HMAS 273757.

#### Notes

Two strains of *T. laevisporum* share identical phenotypic features, and are similar to *T. vinosum* in shape and size of phialides, but the latter produces warted instead of smooth conidia, grows slower in culture [PDA 20–35 mm, on SNA 16–23 mm], has grey-green or darker green colony zonate on CMD, and sporulates later [after 3 wk] in compact pustules on PDA at periphery of the colony^8^. The above morphological divergences reflect in their remote phylogenetic relationship (Fig. 1, MLBP/MPBP/BIPP = 70%/54%/95%).

*Trichoderma hispanicum* and *T. samuelsii* might be related (Fig. 1, MLBP/MPBP/BIPP = 75%/77%/99%), and produce smooth conidia and similar phialides as *T. laevisporum*. However, conidiation pustules of *T. hispanicum* are more compact, and pustules on SNA are formed only at the lateral and distal margin of the Petri dish. *Trichoderma samuelsii* is unique in pyridine-like odor on PDA^9^, which is lacking in *T. laevisporum*.

### **Trichoderma sinokoningii**

**Figure 5:**
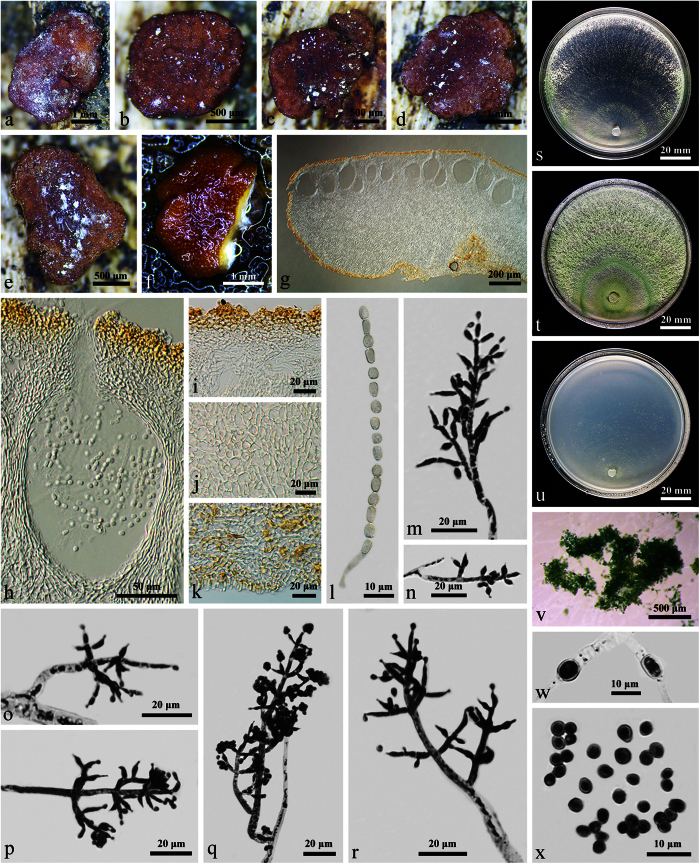
*Trichoderma sinokoningii* (HMAS 271397). (**a–e**) Stromata on natural substrate; (**f**) Rehydrated stroma in 3% KOH; (**g**) Longitudinal section of a stroma; (**h**) Perithecium in section; (**i**) Cortical and subcortical tissues in section; (**j**) Subperithecial tissue in section; (**k**) Stroma base in section; (**l**) Ascus with ascospores; (**m–r**) Conidiophores and phialides (CMD, 5 d); (**s**–**u**) Cultures at 25 C (**s**: CMD, 5 d; **t**: PDA, 7 d; **u**: SNA, 7 d); (**v**) Conidiation pustules (CMD, 7 d); (**w**) Chlamydospores (CMD, 5 d); (**x**) Conidia (CMD, 5 d).

W.T. Qin & W.Y. Zhuang, **sp. nov.** (Fig. 5) Fungal Names: FN570247

#### Typification

China. Henan, Luanchuan, Chongdugou, alt. 1500 m, on twigs, 21 Sep 2013, H.D. Zheng, Z.Q. Zeng & Z.X. Zhu 8849 (holotype HMAS 271397). Ex-type culture HMAS 248727.

#### Etymology

The specific epithet refers to the similarity between the Chinese material and *T. koningii*.

Stromata solitary or scattered, reddish brown, pulvinate, outline circular, oblong or irregular, 2–7 mm diam, 0.7–0.9 mm thick. Surface appearing velvety or smooth with ostiolar openings not visible. Rehydrated stromata not changing color in 3% KOH.

In section, cortical tissue of textura angularis, 16–26 μm thick, not changing color in 3% KOH, cells light brown, thin-walled, 5.3–13.2 × (4.7–)5.3–8 μm (n = 30); subcortical tissue of textura intricata, hyphae hyaline, thin-walled, 3–5.8 μm (n = 30) wide; subperithecial tissue of textura epidermoidea mixed with textura intricata, cells hyaline, thin-walled, (6.5–)8–26 × (6.5–)8–13 μm (n = 30), hyphae hyaline, thin-walled, 5.5–10.5 μm (n = 30) wide; tissue at the base of textura angularis mixed with textura intricata, cells hyaline, thin-walled, 5–10.5 × 5–8 μm (n = 30), hyphae hyaline, thin-walled, 5–9.2 μm (n = 30) wide. Perithecia flask-shaped, 242–289 × 145–210 μm (n = 30); peridium hyaline in lactic acid, not changing color in 3% KOH, (8–)10.5–18.4 μm thick at flanks, 10.5–21 μm thick at the base (n = 30). Ostioles non-papillate, 55–74(–84) μm high, 18–34 μm wide at the apex (n = 30). Asci cylindrical, 82–108(–123) × 5.5–6.8 μm, with a stipe (6–)11–21(–36) μm long (n = 40). Ascospores hyaline, spinulose or verruculose, cells dimorphic, distal cells subglobose to slightly ovoid, 3.8–4.2(–5.2) × 3.2–4.2 μm, l/w 1.0–1.3(–1.6); proximal cells globose, subglobose to nearly wedge-shaped, 4–5.5(–6) × 3–3.5 μm, l/w (1.1–)1.2–1.6(–1.7) (n = 40).

On CMD after 72 h 50–52 mm, mycelium covering the plate after 4 d at 25 C. Colony hyaline with circle outline; aerial hyphae common, faintly zonate, conidiation noted after 24 h at the periphery of the colony, first white, turning green after 48 h. Conidiophores trichoderma-like, more or less symmetrical, with 2–5 whorls arising from the main axis. Phialides lageniform, subulate or slightly swollen in the middle, 6–11(–15) × 2.5–3.5 μm, l/w (1.7–)1.9–4(–4.3), 1.8–3 μm wide at the base (n = 50). Conidia green, globose, subglobose or ellipsoidal, smooth, (2.8–)3–5.5(–6) × (2–)2.8–3(–3.5) μm, l/w 1.0–2.0(–2.5) (n = 40). Chlamydospores noted after 4 d, terminal or intercalary, globose, ellipsoidal, fusoid or rectangular, 7–13 × 7–9.5, l/w 1.0–1.4(–1.9) (n = 40). No distinct odor, no diffusing pigment observed.

On PDA after 72 h 38–40 mm, mycelium covering the plate after 5 d at 25 C. Colony dense with circle outline; aerial hyphae numerous, typically zonate. Conidiation noted after 24 h on or around the inoculation plug. Pustules first white, later turning pale green. Chlamydospores common. A distinct coconut-like odor detected, no diffusing pigment observed.

On SNA after 72 h 20–22 mm, mycelium covering the plate after 8 d at 25 C. Colony hyaline, thin with aerial hyphae sparsely disposed. Conidiation noted after 24 h on or around the plug. No distinct odor, no diffusing pigment observed.

#### Notes

*Trichoderma sinokoningii* gives rise to reddish brown stromata with invisible ostiolar dots, which is typical in the Viride clade. As its closest relative, *T. austrokoningii* differs in smaller stromata [1–2 mm], wider perithecia [175–300 × 120–150 μm], and smaller asci [(65–)75–90(–100) × (3.2–)4.2–5.7(–6.5) μm]. Besides, it produces simple conidiophores bearing whorls of 3 or 4 phialides and much smaller conidia [(2.5–)3.0–3.7(–4.2) × (2.0–)2.2–2.7(–3.2) μm], and lacks chlamydospores and coconut-like odor on PDA^10^. The two species share very low sequence similarity, and there are 26 bp divergences among 921 bp for *RPB2* (97.2%) and 35 bp among 1176 bp for *TEF1-α* (97%).

### **Trichoderma sparsum**

**Figure 6:**
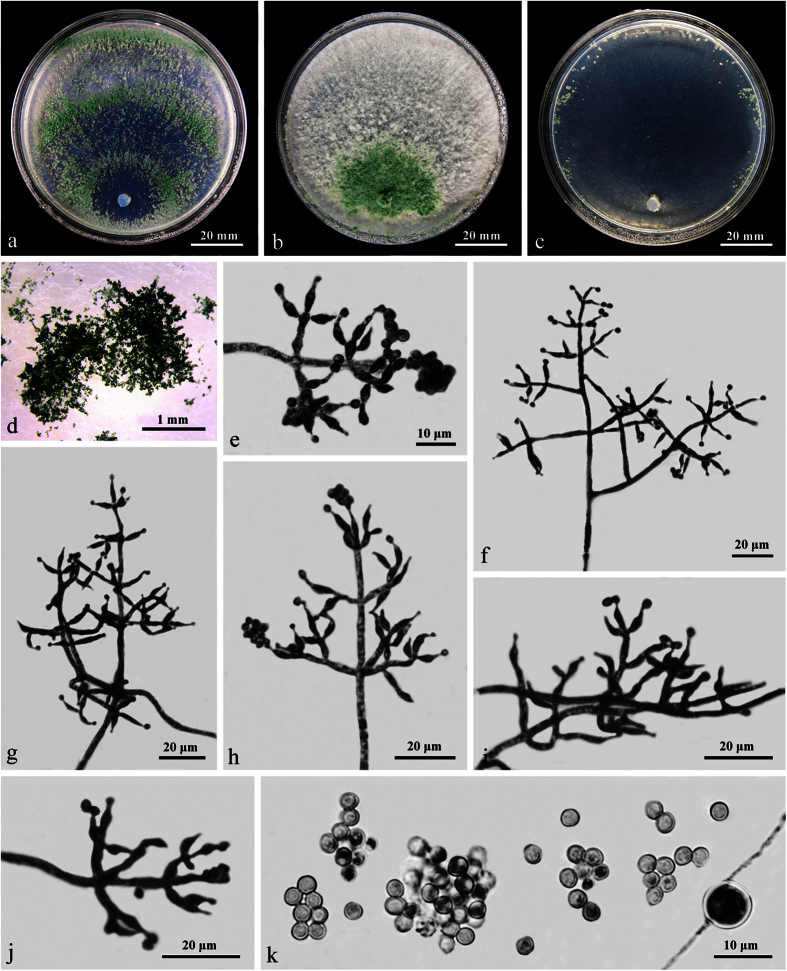
*Trichoderma sparsum* (HMAS 273759). (**a–c**) Cultures after 6 d at 25 C (**a**: CMD; **b**: PDA; **c**: SNA); (**d**) Conidiation pustules (CMD, 8 d); (**e–j**) Conidiophores and phialides (CMD, 4 d); (**k**) Chlamydospores and conidia (CMD, 4 d).

W.T. Qin & W.Y. Zhuang, **sp. nov.** (Fig. 6) Fungal Names: FN570246

#### Typification

China. Hunan, Dongan, Shunhuangshan, alt. 800 m, on twigs, 14 Jul 2015, Z.Q. Zeng 10122 (holotype HMAS 273759).

#### Etymology

The specific epithet refers to its sparse conidiophores and phialides.

On CMD after 72 h mycelium covering the plate at 25 C. Colony hyaline, circular, with distinct concentric zones. Conidiation starting after 3 d, developing abundantly at the edges of the concentric rings; surface becoming downy, floccose or farinose due to conidial heads. Conidiophores formed widely spaced on aerial hyphae, trichoderma-like. Phialides often symmetrical, solitary or commonly divergent in whorls of 2–3(–4), lageniform to subulate, straight or slightly curved, (6–)7–12(–13) × 2–3.2 μm, l/w (2–)2.2–5.5(–7.2), 1.2–2 μm wide at the base (n = 40). Conidia green, mostly globose, subglobose or oval, rare ellipsoidal, smooth, 2.8–3.5(–4.2) × 2.8–3.5 μm, l/w 1.0–1.2(–1.3) (n = 50). Chlamydospores noted after 4 d, terminal or intercalary, globose, ellipsoidal or rectangular, 5–11 × 5–10, l/w 1.0–1.6 (n = 40). No distinct odor, no diffusing pigment observed.

On PDA after 72 h mycelium covering the plate at 25 C. Colony whitish, conspicuously dense, typically not zonate with distinct circular outline and well-defined margin. Aerial hyphae numerous, densely disposed in the centre, thick and branched, mostly radially arranged, making surface becoming hairy. Conidiation starting after 2 d on and around the plug in short minute shrubs, spreading, growing to tufts or pustules, green. No distinct odor, no diffusing pigment observed.

On SNA after 72 h 55 mm and mycelium covering the plate after 4 d at 25 C. Colony hyaline, thin, aerial hyphae loosely arranged, radially. Conidiation noted after 3 d along the margin. No distinct odor, no diffusing pigment observed.

#### Notes

*Trichoderma sparsum* is diagnostic by fast growth, downy, floccose or farinose surface of colonies on CMD and PDA, trichoderma-like conidiophores, and globose or subglobose conidia. It shares common ancestor with *T. rogersonii* and *T. subeffusum* (Fig. 1). However, *T. rogersonii* grows much slower [CMD 38–43 mm, PDA 39–43 mm, SNA 33–41 mm], and the mycelium covered the plate after 5–7 d at 25 C in cultures, and it forms larger chlamydospores [(7–)9–15(–26) μm diam] and larger conidia [(2.7–)3.5–4.5(–5.5) × (2.2–)2.5–3.2(–4.2) μm]^10^. *Trichoderma subeffusum* is among the very few species that sporulate well on CMD but poorly on SNA, and possesses large coilings on colony surface of CMD^19^, which do not exist in *T. sparsum*.

### **Trichoderma sphaerosporum**

**Figure 7:**
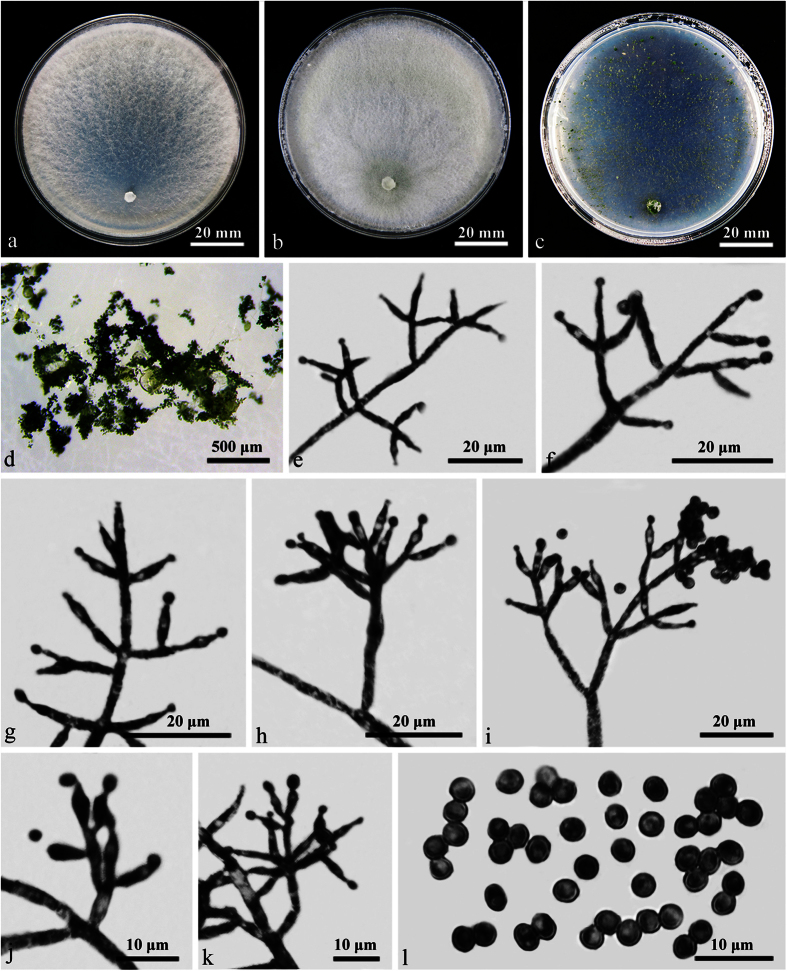
*Trichoderma sphaerosporum* (HMAS 273763). (**a–c**) Cultures at 25 C (**a**: CMD, 14 d; **b**: PDA, 12 d; **c**: SNA, 12 d); (**d**) Conidiation pustules (SNA, 14 d); (**e–k**) Conidiophores and phialides (SNA, 6 d); (**l**) Conidia (SNA, 6 d).

W.T. Qin & W.Y. Zhuang, **sp. nov.** (Fig. 7) Fungal Names: FN570244

#### Typification

China. Hubei, Shennongjia, Qiaonenggou, alt. 1950 m, on twigs, 16 Sep 2014, W.T. Qin, Z.Q. Zeng, K. Chen & H.D. Zheng 9755 (holotype HMAS 273763).

#### Etymology

The specific epithet refers to the globose to subglobose conidia.

On CMD after 72 h 45–46 mm and mycelium covering the plate after 8 d at 25 C. Colony dense, whitish, with a well-defined margin. Aerial hyphae apparent toward the downy or floccose distal margin, becoming fertile. A distinct coconut-like odor detected, no diffusing pigment observed.

On PDA after 72 h 28–30 mm and mycelium covering the plate after 10 d at 25 C. Colony dense, white, macroscopically homogeneous, circle outline. Conidiation noted after 2 d, conidiophores formed widely spaced on abundant aerial hyphae. A distinct coconut-like odor detected, no diffusing pigment observed.

On SNA after 72 h 25–27 mm and mycelium covering the plate after 10 d at 25 C. Colony hyaline, thin, aerial hyphae loosely disposed, radially fan-shaped. Conidiation noted after 3 d at 25 C, slightly more abundant and denser than on CMD and PDA. Conidiophores trichoderma-like, phialides solitary or commonly divergent in whorls of 2–3(–4) from the main axis, lageniform or ampulliform, (6–)6.5–10(–10.5) × 2–2.5(–3) μm, l/w (2.2–)2.5–4.6, 1–2 μm wide at the base (n = 120). Conidia light green to green, mostly globose or subglobose, sometimes oval to ellipsoidal, smooth, (2.7–)2.9–3.8(–4.2) × (2.5–)2.7–3.2 μm, l/w 1.0–1.3(–1.4) (n = 120). No chlamydospores observed. A distinct coconut-like odor detected, no diffusing pigment observed.

#### Other specimens examined

China. Hubei, Shennongjia, Dalongtan, alt. 2000 m, on twigs, 13 Sep 2014, W.T. Qin, Z.Q. Zeng, H.D. Zheng & K. Chen 9479, HMAS 273764; Shennongjia, Dalongtan, alt. 2000 m, on twigs, 13 Sep 2014, H.D. Zheng, Z.Q. Zeng, W.T. Qin & K. Chen 9439, HMAS 273765.

#### Notes

Sharing exactly the same sequences, the three strains of *T. sphaerosporum* form a separate lineage in relation to *T. junci* (Fig. 1). Although their colony morphologies on PDA are similar, *T. junci* produces abundant grey-green tufts or pustules on CMD, and has simple and larger phialides [(6–)8–14(–19) × (2.0–)2.5–3.3(–3.7) μm] and much larger conidia [(3.5–)3.7–4.6(–5.3) × (2.4–)2.5–3.0 μm]^19^.

### **Trichoderma subviride**

**Figure 8:**
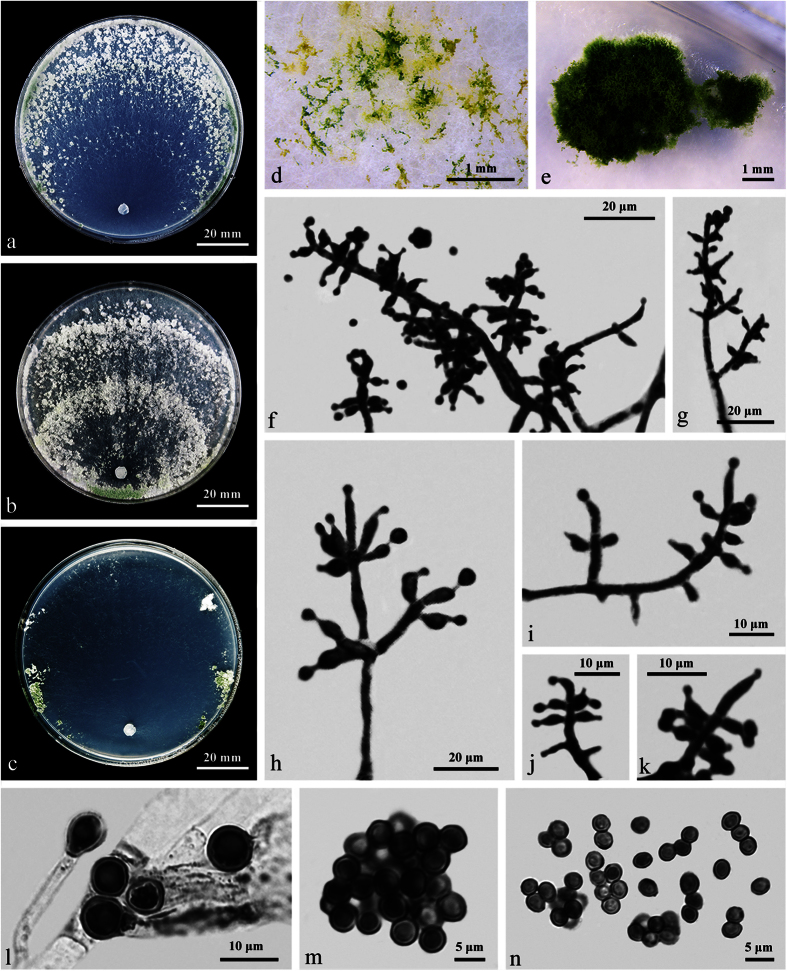
*Trichoderma subviride* (HMAS 273761). (**a–c**) Cultures after 6 d at 25 C (**a**: CMD, **b**: PDA, **c**: SNA); (**d,e**) Conidiation pustules (d: PDA, 15 d; e: SNA, 10 d); (**f–k**) Conidiophores and phialides (SNA, 10 d); (**l**) Chlamydospores (SNA, 10 d); (**m,n**) Conidia (SNA, 10 d).

W.T. Qin & W.Y. Zhuang, **sp. nov.** (Fig. 8) Fungal Names: FN570245

#### Typification

China. Henan, Lingbao, Yanzishan, alt. 1000 m, on twigs, 16 Sep 2013, H.D. Zheng, Z.Q. Zeng & Z.X. Zhu 8658 (holotype HMAS 273761).

#### Etymology

The specific epithet refers to the similarity of the fungus to *T. viride*.

On CMD after 72 h 58–60 mm, mycelium covering the plate after 4 d at 25 C. Colony dense, with well-defined outline, aerial hyphae numerous. Conidiation noted after 2 d in distal areas of the plate, first effuse in minute shrubs, later in numerous minute granules and pustules with granulose or plumose surface, pale green. A distinct coconut-like odor detected, no diffusing pigment observed.

On PDA after 72 h 60–63 mm, mycelium covering the plate after 4 d at 25 C. Colony, dense, aerial hyphae abundant, conidiation noted after 2 d in numerous pale yellow to yellow green squamose granules or tufts and gradually formed two concentric zonate. A distinct coconut-like odor detected, no diffusing pigment observed.

On SNA after 72 h 43–45 mm, mycelium covering the plate after 5 d at 25 C. Colony hyaline, aerial hyphae numerous, conidiation noted after 2 d in the pustules or tufts along the margin, first white, finally pale green. Conidiophores trichoderma-like, phialides mostly symmetrically arranged, solitary or divergent in whorls of 2–4(–6), lageniform or ampulliform, straight or slightly curved or sinuous, (4.5–)5–6.5(–7) × 2–3 μm, l/w (1.4–)1.7–3.3(–3.5), 1.2–2.2 μm wide at the base (n = 80). Conidia green, globose or subglobose, sometimes ellipsoidal, smooth, (2–)2.5–3.8(–4.5) × (2–)2.5–3.2, l/w 1.0–1.5(–1.9) (n = 80). Chlamydospores noted after 8 d, terminal or intercalary, mostly globose or subglobose, ellipsoidal or fusoid, sometimes rectangular, 5–8.5(–9.5) × (4–)5–7.5(–8.5), l/w 1.0–1.7(–1.8) (n = 80). A distinct coconut-like odor detected, no diffusing pigment observed.

### Other specimen examined

China. Henan, Luoyang, Yushan, alt. 1200 m, on twigs, 23 Sep 2013, H.D. Zheng, Z.Q. Zeng & Z.X. Zhu 8878, HMAS 273762.

#### Notes

*Trichoderma subviride* is featured by pale yellow to yellow green conidiation zones with a squamose surface on PDA, which has not been seen in any other known species of the genus. *Trichoderma subviride* was associated with *T. atroviride* and *T. paratroviride* as indicated by the sequence analyses (Fig. 1); however, phialides and conidia are larger in *T. atroviride* [(4–)6–10(–12) × (2.0–)2.3–3.0(–3.3) μm, (2.8–)3.2–4.0(–4.7) × (2.8–)3.0–3.5(–3.8) μm] and *T. paratroviride* [(5.2–)6.2–11(–14) × (2.0–)2.5–3.2(–3.5) μm, (3.0–)3.3–3.7(–4.0) × (3.0–)3.2–3.5(–3.7) μm], and the growth rates of *T. atroviride* [CMD 45–48 mm, PDA 57–62 mm, SNA 34–37 mm] and *T. paratroviride* [CMD 49–62 mm, PDA 54–56 mm, SNA 30–33 mm] are smaller^12^,^19^.

## Discussion

More than 100 recent collections of *Trichoderma* Viride clade from northern and central China were examined, and seven new species are found based on the integrated studies of phenotypic and molecular data. To explore their taxonomic positions, the phylogenetic tree containing all species of the Viride clade was constructed based on analyses of the combined sequences of *RPB2* and *TEF1-α.* The 69 currently known species in this clade clustered together (Fig. 1). Six subclades, Hamatum/Asperellum, Koningii, Neorufum, Rogersonii, Viride and Viridescens, were recognized, which is basically congruent with the results by Jaklitsch and Voglmayr^12^. The seven new species are well-located in the Viride clade, and distributed in Koningii, Neorufum and Rogersonii subclades as well as the unnamed terminal branches; which are clearly distinguishable from any existing species.

Monographic treatments of the *Trichoderma* species with hyaline ascospores were carried out by Jaklitsch^19^ and Jaklitsch & Voglmayr^12^. Hyaline-ascospored species of the genus have been divided into nine clades, Brevicompactum, Deliquescens, Hypocreanum, Longibrachiatum, Polysporum, Psychrophilum, Semiorbis, Stromaticum, and Viride. Their conidia are mostly smooth, and less frequently verruculose, verrucose, tuberculate or warted. Twenty-two species are reported to have ornamented conidia, which are distributed in the Longibrachiatum and Viride clades, of which 18 belong in the Viride clade^8^,^9^,^11^,^22^.

Taxonomy of *Trichoderma* in China dates back to 1895^23^. In more than a century, successive findings have brought the number of the known species of the genus in China up to over 100^17^,^24^,^25^,^26^. Species of the genus are located throughout the country, among which 75 are wood-inhabiting and have been found in Anhui, Fujian, Guangdong, Guangxi, Guizhou, Hainan, Hebei, Henan, Hubei, Hunan, Jiangsu, Jiangxi, Jilin, Liaoning, Shandong, Sichuan, Taiwan, Xizang, Yunnan, and Zhejiang provinces. As to the Viride clade, 13 species are wood-inhabiting among the 22 taxa reported in the country^10^,^27^,^28^,^29^,^30^,^31^,^32^. China is one of the world biodiversity hotspots. *Trichoderma* serves as a good example. The known species of the genus in China occupy 40% of the world records.

Along with increased number of species joining the Viride clade and through the integrated studies on morphology, ecology and phylogeny, our understanding of the group will become more sophisticated and intelligible, reasonable species concepts will be firmly established, and co-relation between morphology and sequence data will be explored. Accumulations of our knowledge of *Trichoderma* will provide useful information for sufficient utilization of fungal resources.

## Materials and Methods

### Isolates and specimens

Specimens examined were collected from Beijing, Henan, Hubei and Hunan provinces, China. Strains were obtained from direct isolation from asexual morphs on the substrates or single-ascospore isolation following the method by Jaklitsch^33^. They were deposited in the fungarium of the Institute of Microbiology, Chinese Academy of Sciences (HMAS).

### Morphological characterization

Methods and morphology were described basically following counterparts by Jaklitsch^33^ and Jaklitsch & Voglmayr^12^. Longitudinal sections of rehydrated stromata were made at a thickness of 8–10 μm using a freezing microtome (YD-1508-III, Yidi Medical Appliance Factory, Jinhua, Zhejiang, China). Colony radius and characteristics on Cornmeal Dextrose agar (CMD), Potato Dextrose agar (PDA) and synthetic low nutrient agar (SNA) were measured and noted. Photographs were taken with a Leica DFC450 digital camera (Wetzlar, Germany) connected to a Leica M125 stereomicroscope (Milton Keynes, UK) for gross morphology and a Zeiss AxioCam MRc 5 digital camera (Jena, Germany) connected to a Zeiss Imager A2 microscope (Göttingen, Germany) for anatomical structures.

*DNA extraction, PCR amplification and sequencing*.—Genomic DNAs were extracted following methods by Wang & Zhuang^34^ from mycelia prepared previously. Two primer pairs, fRPB2-5f and fRPB2-7cr^35^, EF1-728F^36^ and TEF1LLErev^37^ were separately used to amplify fragments of *RPB2* and *TEF1-α*. PCR products were purified with the PCR Product Purification Kit (Biocolor BioScience and Technology Co., Shanghai, China) and then cycle-sequenced using the primer pairs reported by Jaklitsch^33^ on an ABI 3730 XL DNA Sequencer (Applied Biosciences, Foster City, CA, USA) at Beijing Tianyihuiyuan Bioscience and Technology, China. The sequences used in this study are provided in Supplementary Table S1.

### Phylogenetic analyses

To locate the phylogenetic positions of the seven new species, 76 combined *Trichoderma* sequences of *RPB2* and *TEF1-α* were used for analyses, among them *T. danicum* and *T. spinulosum* were outgroup taxa. The sequences were aligned and assembled with BioEdit 7.0.5.3^38^, NEXUS files were subsequently generated by ClustalX 1.83^39^.

Maximum likelihood analysis was carried out with RaxML^40^ with the PHY files generated by Mesquite 2.75^41^. GTRGAMMA was specified as the model. The analysis was run with a rapid bootstrap analysis using a random starting tree. Topological confidence of resulted trees was assessed by bootstrap proportion (BP) with 1000 replicates.

Maximum parsimony analysis was conducted by PAUP 4.0b10^42^ using a heuristic search with tree-bisection-reconnection branch swapping. All characters were treated as unordered and unweighted, gaps were treated as missing data, sequences were auto-increased and Maxtrees was 1000. Topological confidence of resulted trees was tested by bootstrap proportion with 1000 replicates, each with 10 replicates of random addition of taxa.

Bayesian Inference analysis was performed with MrBayes 3.1.2^43^ using Markov ChainMonte Carlo (MCMC) algorithm. Appropriate nucleotide substitution models was determined by MrModeltest 2.3^44^ and the best fit model GTR+I+G was selected by Akaike Information Criterion for the investigated data set. Four MCMC chains (one cold and three heated) were run for one million generations with the trees sampled every 100 generations. The first 25% trees were excluded as the burn-in phase of the analyses, and posterior probability (PP) values were estimated by the 75% remaining trees. Trees were viewed in TreeView 1.6.6^45^.

## Additional Information

**How to cite this article**: Read: Qin, W.T. and Zhuang, W.Y. Seven wood-inhabiting new species of the genus *Trichoderma* (Fungi, Ascomycota) in Viride clade. *Sci. Rep.* **6**, 27074; doi: 10.1038/srep27074 (2016).

## Supplementary Material

Supplementary Information
