# Corrigendum: Seven wood-inhabiting new species of the genus *Trichoderma* (Fungi, Ascomycota) in Viride clade

**DOI:** 10.1038/srep30100

**Published:** 2016-07-25

**Authors:** Wen-Tao Qin, Wen-Ying Zhuang

Scientific Reports 6: Article number: 2707410.1038/srep27074; published online: 06 01 2016; updated: 07 25 2016

This Article contains errors in the Author Contributions Statement.

“Conceived and designed the experiments: W.Y.Z. and W.T.Q. Performed the experiments, analyzed the data and wrote the paper: W.T.Q. Revised and approved the final version of the paper: W.Y.Z”.

should read:

“Conceived and designed the experiments: W.Y.Z. Performed the experiments, analyzed the data and wrote the paper: W.T.Q. Revised the manuscript and approved the final version of the paper: W.Y.Z”. 

