# Supplementary material for: Seven wood-inhabiting new species of the genus Trichoderma (Fungi, Ascomycota) in Viride clade
Source: Sci Rep. 2016 Jun 1;6:27074. doi: 10.1038/srep27074 (PMC4888246; doi:10.1038/srep27074)
Supplement: Supplementary Information [file srep27074-s1.pdf]

Seven wood-inhabiting new species of the genus *Trichoderma* (Fungi, Ascomycota) in Viride clade

Wentao Qin[1, 2], Wenying Zhuang[1*]

[1]State Key Laboratory of Mycology, Institute of Microbiology, Chinese Academy of Sciences, Beijing 100101, P.R. China

[2]University of Chinese Academy of Sciences, Beijing 100049, P.R. China

[*]Corresponding author. E-mail: zhuangwy@im.ac.cn

Supplementary Table S1 Materials used for phylogenetic analyses

Species	Strain	GenBank accession no.	
		RPB2	*TEF1*
T. aeroaquaticum K. Yamag., Tsurumi, Chuaseehar. & Nakagiri	NBRC 108031	AB646529	AB646533
T. albofulvopsis W.T. Qin & W.Y. Zhuang	HMAS 273760	**KU529138**[*]	**KU529127**
T. albofulvum (Berk. & Broome) Jaklitsch & Voglmayr	GJS 01-265	KR094870	DQ835494
T. appalachiense Samuels & Jaklitsch	GJS 00-67	–	DQ307502
T. asperelloides Samuels	GJS 99-6	GU198271	DQ109550
T. asperellum Samuels, Lieckf. & Nirenberg	GJS 05-328	EU248614	EU248627
T. atroviride P. Karst.	CBS 119499	FJ860518	FJ860611
T. austrokoningii Samuels & Druzhin.	CBS 247.63	FJ442772	–
	Hypo 498	–	KJ665433
T. caerulescens (Jaklitsch & Voglmayr) Jaklitsch & Voglmayr	S195	JN715604	JN715621
T. caribbaeum Samuels & Schroers	GJS 97-3	KJ665246	KJ665443
T. composticola Samuels & Jaklitsch	CBS 439.95	KC285753	AY937413
T. danicum Jaklitsch	CBS 121273	FJ860534	FJ860634
T. densum W.T. Qin & W.Y. Zhuang	HMAS 273758	**KU529137**	**KU529126**
T. dingleyae Samuels & Dodd	GJS 99-105	EU341803	DQ289008
T. dorotheae Samuels & Dodd	GJS 99-202	EU248602	DQ307536
T. eijii C.S. Kim & N. Maek.	TUFC 100002	JX238484	JX684011
T. erinaceum Bissett, C.P. Kubicek & Szakács	DIS 7	EU248604	DQ109547
T. evansii Samuels	DIS 341HI	EU883558	EU883566
T. flaviconidium (P. Chaverri, Druzhin. & Samuels) Jaklitsch & Voglmayr	GJS 99-49	EU883557	DQ020001
T. gamsii Samuels & Druzhin.	S488	KJ665270	JN715613
T. hamatum (Bonord.) Bainier	Hypo 647	KJ665274	KJ665513
T. hispanicum (Jaklitsch & Voglmayr) Jaklitsch & Voglmayr	S453	JN715600	JN715659
T. intricatum Samuels & Dodd	GJS 02-78	EU241505	EU248630
T. istrianum Jaklitsch & Voglmayr	S123	KJ665280	KJ665521
T. junci Jaklitsch	CBS 120926	FJ860540	FJ860641
T. koningii Oudem.	S227	JN715609	KC285596
T. koningiopsis Samuels, C. Suárez & H.C. Evans	GJS 04-199	FJ442789	FJ463268
T. laevisporum W.T. Qin & W.Y. Zhuang	HMAS 273756	**KU529139**	**KU529128**
	HMAS 273757	**KU529140**	**KU529129**
T. lieckfeldtiae Samuels	GJS 00-15	EU883561	DQ109543
T. martiale Samuels	GJS 04-40	EU248597	EU248618
T. matsushimae (Abdullah & J. Webster) K. Yamag., Tsurumi, Chuaseehar. & Nakagiri	IMI 266915	–	AB646534
T. neokoningii Samuels & Soberanis	GJS 04-216	KJ665318	KJ665620
T. neorufoides Jaklitsch	CPK 1904	FJ860554	FJ860658
T. neorufum (Samuels, Dodd & Lieckf.) Jaklitsch & Voglmayr	CBS 119498	FJ860550	FJ860653
T. neosinense Samuels & Jaklitsch	GJS 94-11	KC285777	KJ665624
T. nothescens Samuels & Jaklitsch	GJS 99-142	EU241498	DQ307512
T. ochroleucum (Berk. & Ravenel) Jaklitsch & Voglmayr	CBS 119502	FJ860556	FJ860659
T. olivascens Jaklitsch, Samuels & Voglmayr	S34	KC285751	KC285615

T. ovalisporum Samuels & Schroers	GJS 04-113	FJ442781	FJ463281
T. pararogersonii Jaklitsch & Voglmayr	S301	KJ665320	KJ665625
T. paratroviride Jaklitsch & Voglmayr	S385	KJ665321	KJ665627
T. paraviridescens Jaklitsch, Samuels & Voglmayr	S122	KC285764	KC285671
T. paucisporum Samuels, C. Suárez & K. Solis	GJS 03-69	EU883560	DQ109541
T. petersenii Samuels, Dodd & Schroers	CBS 119507	FJ860568	FJ860670
T. pezizoides (Berk. & Broome) Samuels, Jaklitsch & Voglmayr	GJS 01-257	EU248608	AY937438
T. pubescens Bissett	GJS 01-207	FJ150768	EU856304
T. rogersonii Samuels	CBS 119503	FJ860583	FJ860690
T. samuelsii Jaklitsch & Voglmayr	S42	JN715598	JN715652
T. scalesiae Samuels & H.C. Evans	GJS 03-74	EU252007	DQ841726
T. sempervirentis Jaklitsch & Voglmayr	S599	KC285755	KC285632
T. sinokoningii W.T. Qin & W.Y. Zhuang	HMAS 271397	**KU529141**	**KU529130**
T. songyi M.S. Park, S.Y. Oh & Y.W. Lim	SFC20130926-S001	KJ636518	KJ636525
T. sparsum W.T. Qin & W.Y. Zhuang	HMAS 273759	**KU529147**	**KU529136**
T. sphaerosporum W.T. Qin & W.Y. Zhuang	HMAS 273765	**KU529144**	**KU529133**
	HMAS 273764	**KU529146**	**KU529135**
	HMAS 273763	**KU529145**	**KU529134**
T. spinulosum (Fuckel) Jaklitsch & Voglmayr	CBS 121272	FJ860590	FJ860700
T. stilbohypoxyli Samuels & Schroers	CBS 119501	FJ860593	FJ860703
T. strigosellum C.A. Lopez-Q, W. Gams, T. Boekhout & I. Druzhinina	GJS 05-02	EU248607	EU248631
T. strigosum Bissett	DAOM 166121	AF545556	AY937442
T. subeffusum Jaklitsch	CBS 120929	FJ860597	FJ860707
T. subviride W.T. Qin & W.Y. Zhuang	HMAS 273761	**KU529142**	**KU529131**
	HMAS 273762	**KU529143**	**KU529132**
T. taiwanense Samuels & M.L. Wu	CPK 416	JN715608	–
	GJS 95-93	–	DQ284973
T. theobromicola Samuels & H.C. Evans	Dis 376f	FJ150786	EU856322
T. trixiae Samuels & Jaklitsch	GJS 92-11	KC285771	DQ307524
T. valdunense Jaklitsch	CBS 120923	FJ860605	FJ860717
T. vinosum Samuels	GJS 99-158	KC285779	AY376047
T. viridarium Jaklitsch, Samuels & Voglmayr	S136	KC285760	KC285658
T. viride Pers.	CBS 119325	EU711362	DQ672615
T. viridescens (A.S. Horne & H.S. Will.) Jaklitsch & Samuels	S452	KC285758	KC285646
T. viridialbum Jaklitsch, Samuels & Voglmayr	S250	KC285774	KC285706
T. virilente Jaklitsch & Voglmayr	S520	KC285769	KC285695
T. voglmayrii Jaklitsch	CBS 117710	DQ086151	DQ086147
T. yunnanense Z.F. Yu & K.Q. Zhang	CBS 121219	GU198274	GU198243

[*]Numbers in boldface indicate newly submitted sequences.
